# Cell-in-Cell Phenomena in Wall-Less Bacteria: Is It Possible?

**DOI:** 10.3390/ijms23084345

**Published:** 2022-04-14

**Authors:** Innokentii E. Vishnyakov

**Affiliations:** Institute of Cytology, Russian Academy of Sciences, 194064 St. Petersburg, Russia; innvish@gmail.com; Tel.: +7-812-297-0328

**Keywords:** cell-in-cell, wall-less bacteria, mycoplasma, membrane fusion, conjugation, exchange of genetic material

## Abstract

This work describes curious structures formed by the mainly phytopathogenic mycoplasma *Acholeplasma laidlawii*, as well as the human pathogen *Ureaplasma parvum* cells which resemble cell-in-cell structures of higher eukaryotes and protists. The probable significance of such structures for the mycoplasma cell is discussed. The possibility of their formation in nature and their potential role in the transformation of genetic material, for example, by maintaining (on the one hand) the stability of the genome in the line of generations during asexual reproduction or (on the other hand) the genome plasticity, are substantiated. It should be especially noted that all the arguments presented are based only on morphological data. However, closer attention to unusual structures, the existence of which was shown by electron microscopy images in this case, may prompt researchers to analyze their data more carefully and find something rare and non-trivial among seemingly trivial things. If it is proven by additional methods that cell-in-cell structures can indeed be formed by prokaryotes without a cell wall, this phenomenon may acquire general biological significance.

## 1. Introduction

Interesting cases of cell–cell fusions and cell-in-cell phenomena in both higher eukaryotes and protists have been discussed recently [1]. The formation of cell-in-cell structures has been shown both for healthy human cells, in particular, for liver cells [2], and for cancer cells [3]. The unstable nature of the latter can additionally contribute to the acceleration of their fusogenic potential and an increase in cell–cell fusions [4]. Normal cell fusions between healthy cells are considered to occur mainly due to the so-called “fusogenic proteins”, or “fusogens”; for the most part, these are transmembrane proteins [5].

The “polymorphism” of cell fusions and multinuclearity of protists were independently and repeatedly observed in various phylogenetically distant macrotaxons [1]. The authors, after thorough analysis of a large amount of factual material, conclude that the complete or partial fusion of vegetative cells—somatogamy—is common in the protist life cycles, and the ability for fusion is one of the fundamental features of all living biological objects at the cellular level of organization which provides them with the mainstream strategy of survival and biodiversity maintenance.

Most bacteria, by their nature, cannot be capable of cell–cell fusions and the formation of cell-in-cell structures, since they have a cell wall that prevents cells (membranes) from fusing with each other. However, bacteria have a conjugation process [6], which allows the exchange of genetic material with each other, including with the aim of acquiring beneficial properties by recipient cells, such as antibiotic resistance [7].

Mycoplasmas (class *Mollicutes*) do not have a cell wall but only “traditional” lipid bilayer, and are characterized by sizes close to the theoretically calculated minimum for living systems capable of independent reproduction [8]. Researchers have long been interested in how mycoplasmas, on the one hand, maintain the stability of the genome through a series of generations, and, on the other hand, demonstrate a high level of its variability. Recently, the horizontal gene transfer was shown for mycoplasmas, and it was demonstrated that the exchange of genetic material between their cells is of a both classical and non-classical nature [9].

Additionally, cases of fusion of mycoplasma membranes with cells of higher eukaryotes have been described [10,11,12,13,14]. These cases, in general, are rare. For example, only a small fraction of the T-lymphocyte population (up to 10% of the cells) is capable of fusion with mycoplasmas [10]. Choline-containing lipids of *Mycoplasma fermentans* were shown to participate in the adhesion to the surface of eukaryotic cells and the mycoplasma cell fusion with them [13]. Such fusion induces cytokine secretion by cells of the immune system. Interactions of *Acholeplasma laidlawii* cells with mouse spleen lymphocytes can lead to their fusion and exchange of membrane components between mycoplasmas and eukaryotic cells [15]. The potential for fusion of mycoplasma membranes with the membranes of immune cells now is used to produce anti-mycoplasma vaccines [14], and can also be effective to create vectors for the delivery of high-molecular weight soluble substances inside the eukaryotic cell [16].

Moreover, several studies have shown the ability of mycoplasma cells to fuse with each other, being induced from the outside, for example, by the addition of polyethylene glycol [17]. In this case, interspecific hybrids could form, among other things. In addition, a case of the formation of giant *A. laidlawii* cells was described [18], probably due to cell fusion or lagging of the division process from nucleoid replication. In the same work, it was shown that group 1 and group 2 mycoplasma viruses (bacteriophages) can promote the intensification of the formation of giant cells.

Together, these findings prompted us to conduct a large-scale screening of available mycoplasma preparations for the presence of cellular structures resembling cell-in-cell objects of eukaryotes or protists. In this work, there are interesting cases among the *A. laidlawii* PG8a and *Ureaplasma parvum* ser. 3 cells, morphologically resembling cell–cell fusions and cell-in-cell phenomena revealed in higher eukaryotes and protists are presented for the viewer’s judgment and discussion.

This note does not pretend to be some kind of unique discovery. Its task is to induce thoughts, move a little aside from the standard research schemes and carefully developed design of experiments. It is about trying from time-to-time to look at the object of study more broadly. Perhaps some mechanisms occupy a more global place among living matter, and can be found in representatives of even different kingdoms.

## 2. Results

**Figure 1:**
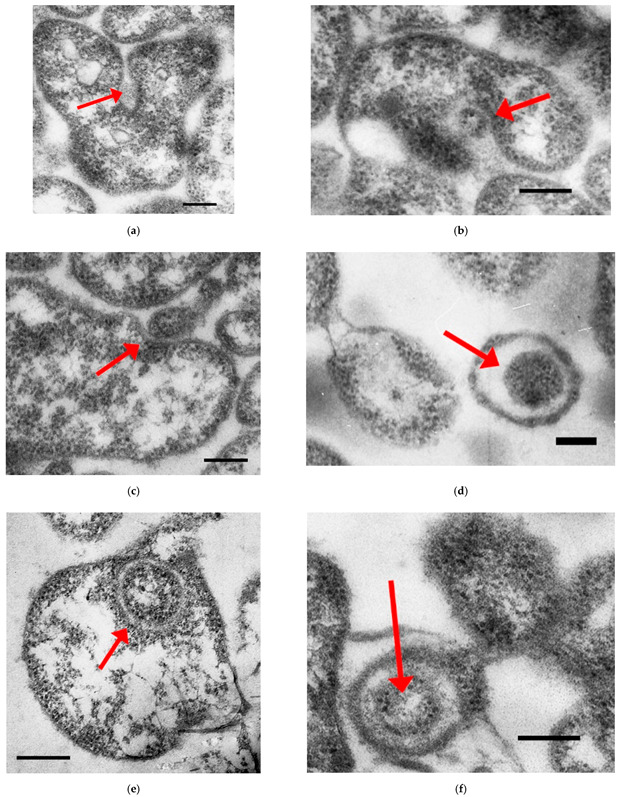
Electron microscopy images of *Acholeplasma laidlawii* PG8a cells. Red arrows point at (**a**) the probable membrane invagination and absorption of some material of low electron density but granular consistency; (**b**) the probable membrane invagination and absorption of a structure resembling the extracellular vesicles of *A. laidlawii*; (**c**) an initial moment of the probable membrane invagination and absorption of a structure resembling the mini-bodies (mini-cells) of *A. laidlawii*; (**d**– **f**) the probable cell-in-cell structures of *A. laidlawii*. Scale bars: 200 nm.

When analyzing the cells of the mainly plant pathogen *A. laidlawii* grown at the optimum temperature of cultivation (+37 °C), 40 fields of view at a magnification of 12,500× were investigated. In the case of the mycoplasma culture subjected to heat shock (+42 °C), 38 fields of view were selected for the analysis. Figure 1 shows electron micrographs of ultrathin sections of *A. laidlawii* cells.

In several cases, patterns were observed that resemble the absorption by mycoplasma cells of some material of low electron density but granular consistency (Figure 1a), or structures similar to extracellular vesicles (Figure 1b), as well as cells large enough for being the normal (vegetative) cells of *A. laidlawii* (about 200–300 nm in diameter, Figure 1c–f). In the latter case, structures resembling smaller mycoplasma cells were located inside the larger cells (500–800 nm in diameter). During the speculative process of probable absorption, a phenomenon similar to the invagination of membranes into the interior of the recipient cell occurs, reminiscent of the initial stages of phagocytosis and vacuolization (Figure 1a–c). This is totally not typical for bacteria, moreover, of such a small size.

Interestingly, the above-described unusual objects have been observed only during optimal temperature growth conditions. Under heat shock, when the formation of *A. laidlawii* mini-cells (mini-bodies) and vesicles should be intensified [19], and cells as a whole should be involved in stress response, such structures, according to our data, have not been found. However, it should be noted that even under optimal growth conditions, this seems to be a very rare phenomenon (9 objects per 870 cells). This could be attributed to sample preparation artifacts, but the presence of an electron-dense fibrillar material between the internalized (donor) and the host (recipient) cells excludes an accidental entry of a smaller cell into a large one. In general, this may indicate the native character of the observed phenomenon.

**Figure 2:**
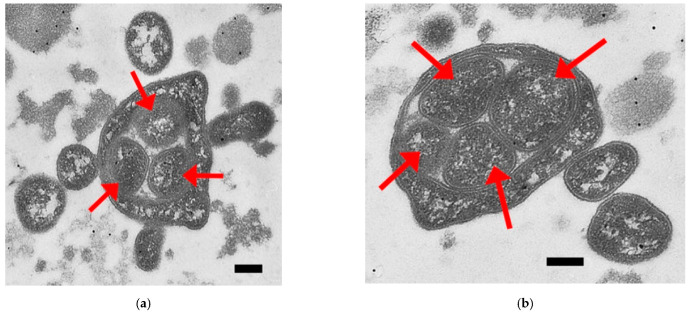
Electron microscopy images of *Ureaplasma parvum* ser. 3 cells. Red arrows point at the probable cell-in-cell structures of *U. parvum*; (**a**) three cells of *U. parvum* in one huge ureaplasma “cell envelope”; (**b**) four cells of *U. parvum* in one huge ureaplasma “cell envelope”, and one of them (left) seems to lose its bilayer. Scale bars: 200 nm.

When working with pathogenic for humans *U. parvum* ser. 3 mycoplasma, about 150 fields of view have been analyzed. Even more interesting objects were observed for *U. parvum* compared to *A. laidlawii* (Figure 2).

Rarely (7 objects per 1021 cells), *U. parvum* cells of a smaller size (300–500 nm in diameter) appeared to be enclosed in a cavity (vacuole?) inside a larger cell (with size ~1 µm or more in diameter), as in a kind of “envelope” or “bag”. The cells inside the “envelope” cavity have clear boundaries, which resemble a classic two-layer cytoplasmic membrane with a distance between layers of about 10 nm, similar in structure to the membrane of the host cell, which contains small cells in itself, as in a “bag”. At the same time, the presence of certain strands of electron-dense material in the cavity between the “cell-bag” and the contents of this “bag” was also well observed. As the micrograph in Figure 2b shows, one of the inner cells seems to have partially lost the bilayer. This may point to the rather natural origin of the phenomenon; at least, it does not resemble an artifact of sample preparation. Of course, there is always a risk of incorrect interpretation of the image, when the plane of the passing section is ignored, or the high level of polymorphism of mycoplasmas, such as microorganisms without a cell wall, is not taken into account.

## 3. Discussion

Can cell–cell fusions and cell-in-cell phenomena be global in nature? Can they persist in such different biological objects as the cells of higher eukaryotes, protists and bacteria without a cell wall? In a recent review [1], the cell-in-cell structures of higher eukaryotes and protists have been compared, and the likelihood of a deep antiquity of this phenomenon has been discussed. The present work provides us with wall-less bacteria examples that are unusual from a morphological point of view, and resemble cell-in-cell structures of higher eukaryotes and protists. It concerns rather dissimilar mycoplasmas—the phytopathogen *A. laidlawii* [20], which has also been found in animals, and may even live autonomously in the environment, and the obligate human parasite *U. parvum* [21]. Interestingly, the former possesses the key division protein FtsZ [22], as well as the small heat shock protein IbpA, which is responsible for the multifaceted response to stress from the very first few minutes [23], and the latter has neither one nor the other [24,25]. At the same time, both mycoplasmas do not have a motility apparatus and are quite polymorphous.

The probable mycoplasmal cell-in-cell structures do not resemble cell–cell fusions, as, for example, in the case of fusion of membrane vesicles with mycoplasma cells described in that paper [26]. It rather resembles entosis, when cell–cell invasion and retarded lysis of internalized cell occurs [27], or emperipolesis, when the engulfed cell can move inside the host cell for some time and even exit it [28]. Moreover, cases of not only destruction of internalized cells, but also their proliferation within cancer cells, have been described [29]. This may be similar to what we see in the case of *U. parvum*. 

It should be especially noted that the objects presented in the work look rather rare among the two species examined. No reports on such structures have been found for other mycoplasmas in literature. If we add to this the polymorphism of mycoplasmas, probable artifacts of sample preparation, the plane of passage of sections, then it is not surprising that such things do not attract the attention of researchers.

One can fantasize a bit about the potential functional meaning of having such structures, at least for wall-less bacteria. Considering the presence of ICEs (**i** ntegrative **c** onjugative **e** lements, [9]) and other mechanisms which provide genetic recombination in the mycoplasma cell [30], one could speculate on the presence of some additional mechanisms that can maintain, due to cell-in-cell structures, the genetic stability of mycoplasmal populations/provide additional flexibility of individuals. For example, ICEs could facilitate an intensive exchange of large regions of nucleoids which are enclosed in a common space after the penetration of one mycoplasma cell into another and the following destruction of its membrane. 

The genomes of a number of mycoplasmas contain ICEs [9,31,32,33]. Functional ICE in at least one partner cell can provide **m** ycoplasma **c** hromosomal **t** ransfer (MCT), an unconventional mechanism, which is involved in the horizontal acquisition of small and large chromosomal fragments from any part of the donor genome [34]. MCT results in progenies composed of an infinitive variety of mosaic genomes. Due to ICEs, the time scale of the mutational pathway leading to high-level of antimicrobial resistance can be readily compressed into a single conjugative step [35]. In *Mycoplasma agalactiae*, it resulted in up to 17% of the genome being exchanged [36]. Intriguingly, due to ICEs, mycoplasmas have retained sexual competence, a trait that may prevent them from genome stasis and contribute to adaptation to new hosts [37]. It is curious if a larger-scale exchange of genetic material (due to ICEs, MCT and cell-in-cell structures) can occur in wall-less prokaryotes, comparable in impact to the genetic recombination of chromosomes during meiosis in eukaryotes. It could be an opposition to Muller’s ratchet, a process that jeopardizes evolutionary longevity of small populations [38]. The rarity of such phenomena also logically fits with this theoretical explanation: too frequent exchanges of large sites could, on the contrary, increase the instability of small mycoplasma genomes. 

Despite the rare occurrence, the speculated events may be of potential interest. It can affect the stability and plasticity of genomes of pathogenic mycoplasmas, including *U. parvum*, or give greater stress resistance to such wall-less prokaryotes as phytoplasmas, close relatives of *A. laidlawii*. This could help bacteria to escape from the host protective systems, medications or antibacterial preparations used for plants treatment.

## 4. Materials and Methods

*Acholeplasma laidlawii* PG8a and *Ureaplasma parvum* ser. 3 cells were cultivated in modified PPLO medium [39] with addition of 0.2% glucose (A.L.) or 0.05% urea (U.P.) until the color of the medium changes to yellow (A.L., approximately 24 h of cultivation) or crimson (U.P., approximately 18 h of cultivation). An aliquot of the *A. laidlawii* PG8a cells suspension was exposed to heat shock (42 °C) for 90 min and then the cells were cultivated during optimal temperature again (37 °C) for 90 min.

*A. laidlawii* PG8a cells were fixed by adding glutaraldehyde right in the suspension of microorganisms to final concentration of 2.5% for 30 min. The cells were collected by centrifugation for 10 min at 10,000 rpm. Supernatant was discarded. The pellet was treated with water solution of 1% OsO_4_ (30 min), dehydrated with increasing ethanol concentrations (70%, 96%) and acetone (100%), impregnated, embedded into Epon-Araldite mixture in gelatin capsules and polymerized for 2 days at 60 °C.

*U. parvum* ser. 3 cells were fixed by adding formaldehyde and glutaraldehyde right in the suspension of microorganisms to final concentrations 2% and 0.1%, respectively. Cells fixed at room temperature for several hours were collected by centrifugation (10,000 rpm, 10 min, 4 °C). The pellet was dehydrated with ethanol increasing concentrations (70%, 96%) and embedded into the LR-White resin (Polyscience, Inc., Warrington, PA, USA) in gelatin capsules. LR-White was polymerized at 52 °C.

Ultrathin sections were prepared by using a microtome Ultratome III 8800 (LKB, Bromma, Sweden), stained with alcoholic uranyl acetate and lead citrate (2–5 min of exposition each) and visualized under electron microscopes Libra 120 (Carl Zeiss, Oberkochen, Germany, A.L.) or JEM-1200 EX (JEOL, Tokyo, Japan, U.P.) at the magnification 10,000–16,000×. 

The difference in the methods of preparing the material is due to the fact that it was prepared at different times for unrelated works [19,40].

## Data Availability

Not applicable.
